# Expansion of the RNAStructuromeDB to include secondary structural data spanning the human protein-coding transcriptome

**DOI:** 10.1038/s41598-022-18699-3

**Published:** 2022-08-25

**Authors:** Warren B. Rouse, Collin A. O’Leary, Nicholas J. Booher, Walter N. Moss

**Affiliations:** 1 Roy J. Carver Department of Biophysics, Biochemistry and Molecular Biology, Iowa State University, Ames, IA 50011 USA; 2 Infrastructure and Research IT Services, Iowa State University, Ames, IA 50011 USA

**Keywords:** RNA, Biochemistry, Computational biology and bioinformatics, Gene expression, Gene regulation, Genomics

## Abstract

RNA plays vital functional roles in almost every component of biology, and these functional roles are often influenced by its folding into secondary and tertiary structures. An important role of RNA secondary structure is in maintaining proper gene regulation; therefore, making accurate predictions of the structures involved in these processes is important. In this study, we have expanded on our previous work that led to the creation of the RNAStructuromeDB. Unlike this previous study that analyzed the human genome at low resolution, we have now scanned the protein-coding human transcriptome at high (single nt) resolution. This provides more robust structure predictions for over 100,000 isoforms of known protein-coding genes. Notably, we also utilize the motif identification tool, ScanFold, to model structures with high propensity for ordered/evolved stability. All data have been uploaded to the RNAStructuromeDB, allowing for easy searching of transcripts, visualization of data tracks (via the Integrative Genomics Viewer or IGV), and download of ScanFold data—including unique highly-ordered motifs. Herein, we provide an example analysis of *MAT2A* to demonstrate the utility of ScanFold at finding known and novel secondary structures, highlighting regions of potential functionality, and guiding generation of functional hypotheses through use of the data.

## Introduction

RNA is a fundamental biomolecule that has importance in all forms of life. Historically, the main function of RNA was believed to be its role as an intermediary, carrying genetic information from DNA to proteins^1,2^; however, this limited conception of the roles of RNA has been upended. Since the advent of high-throughput sequencing in the early 2000s, it is now known that ~70–90 percent of human genomic DNA is transcribed into RNA. Only ~1–3 percent of the genome, however, contains protein coding sequence^3^. While some of the noncoding (nc)RNA transcribed by the cell may be “junk RNA”, many ncRNAs (e.g., long (l)ncRNA and micro (mi)RNA) are functional. Furthermore, RNA structure has roles in the biology of intronic sequences (e.g., in modulating alternative splicing) and in the 5′ and 3′ untranslated regions (UTRs) of mRNAs where it plays regulatory roles^4^.

The UTRs of mRNAs, especially 3'UTRs, are hubs of post-transcriptional regulatory control. Many discrete structured motifs can act in concert to control the stability, localization, and translation of the associated mRNA^5–9^. Interestingly, longer average 3′UTRs lengths are associated with increasing organismal complexity, due to the increased regulation conferred by the 3′UTR and the diversification of resulting protein production^10^. Structures present in the 5′UTR can affect mRNA translation by controlling ribosomal recruitment (e.g., IRES), or by occluding or presenting important trans-acting factor binding sites^11^. Finally, structures present in the coding sequence (CDS) of mRNAs may act as functional, post-transcriptional regulatory motifs (e.g., structures that cause ribosomal pausing, ribosomal frame-shifting, miRNA binding regions, etc.), but they appear to occur less frequently than in the 3′UTR, potentially due to evolutionary constraints to maintain codon and amino acid sequence order^4,8^.

Significantly, dysregulation of RNA structure can lead to a plethora of different diseases: e.g., cancer, neurodegenerative disease, and many others^12–15^. Thus, gaining structural information on mRNA is not only important to basic research, but also provides knowledge that is applicable to human health (e.g., in aiding in the design of RNA-targeting small molecule or antisense oligonucleotide drugs^16–19^). This is especially important for disease causing synonymous mutations that have potential to alter structure without having an effect on the amino acid sequence. This provided partial impetus for our original genome-wide scans contained within the RNAStructuromeDB^20^. Here, we used the RNAfold program^21^ to make structural predictions across every nucleotide of the human hg38 genome using a sliding window approach. A 120 nt analysis window was slid across the genome using a 40 nt step size and the minimum free energy (MFE) of folding (the change in Gibbs folding energy; ΔG) was predicted alongside its associated secondary structure. To assess structure/function propensity, Clote’s method for calculating a thermodynamic z-score^22^ was used to compare the natively ordered RNA sequence to matched randomized sequences^20^. This z-score metric indicates unusual stability of the ordered sequence vs. what one would predict based on the nucleotide content: i.e. the sequence has apparent evolutionary ordering for stable structure^22^.

While the initial human genome scans in the RNAStructuromeDB^20^ are comprehensive, spanning all coding and noncoding regions, they suffer from several limitations. The sliding windows used a large (40 nt) step size that likely led to windows that “cut into” structured regions and did not fully span local domains. Also, while spanning all nucleotides in a gene’s longest pre-mRNA isoform, mature isoforms were not accounted for in the original analysis. Notably, junctions formed by alternative splicing and different length UTRs are missing from the RNAStructuromeDB. An additional limitation is that multiple alternative structure models are possible for each nucleotide, as each was originally only spanned by three analysis windows. This latter limitation is a common feature of sliding window structural analyses, which motivated our development of the ScanFold algorithm^20,23,24^.

ScanFold utilizes a scanning analysis window as a first step to define the local thermodynamic landscape of long RNA transcripts and highlights regions of unusual thermodynamic stability^23,25,26^. This is similar to how we originally scanned the genome but with additional metrics and an extra step to define *unique* structures. This is accomplished via two separate stages, ScanFold-Scan and ScanFold-Fold. First, in ScanFold-Scan, a small scanning analysis window moves along the transcript at regular intervals and calculates local thermodynamic metrics including the MFE and z-score (as before); as well as additional metrics derived from the partition function calculation^27,28^. In the second step, ScanFold-Fold generates a single consensus structure model built from base pairs that reoccur across low z-score analysis windows. The resulting structure model is formed from base pairs that have the greatest bias toward ordered stability and likely functionality^23,24^. These low z-score base paired nucleotides have been found to correlate with low SHAPE probing reactivities, high pairing probabilities^24,29^, and structures solved by both crystallography^30^ and cryo-EM^31^—highlighting ScanFold’s ability to accurately detect highly structured local regions. Additional evidence of its utility is in its ability to detect base pairs that often show significant sequence covariation (correlated mutations) across multiple species.

ScanFold has been used to study several medically significant human mRNAs^32,33^ as well as the genomes of Zika, HIV, Herpesviridae, and (most recently) SARS-CoV-2^24,29,34^. In the analysis of SARS-CoV-2, ScanFold predicted structures, particularly those with significantly negative z-scores, showed high agreement with a myriad of RNA structure probing data sets^29^. Additionally, incorporation of experimentally derived probing data into ScanFold predictions did not significantly alter trends in the z-score metric^29^. This indicates that ScanFold is able to home in on significantly stable regions and produce accurate structural models in these regions with or without experimentally derived probing data. In all targets, ScanFold was not only able to recapitulate known structural motifs, but also deduce novel ones that showed evidence of significant covariation. This not only confirms the ScanFold modeled base pairs, but also their likely functionality. With the enhanced abilities of this program, we revisited human targets by scanning the protein-coding transcriptome.

We applied ScanFold to all human mRNAs (100,552 transcripts isoforms of the 20,342 protein-coding genes). With this more targeted analysis, we used a single nucleotide step and a 120 nt window size. Thus, rather than being spanned by 3 windows (as in our original genome-wide analysis), almost every nucleotide in the transcriptome is spanned by *at least* 120 analysis windows—in cases where isoforms have common sequences, nucleotides can be spanned by many more analysis windows. This enhanced dataset, containing structure scans and ScanFold-Fold models of exceptionally stable motifs, has been uploaded to the RNAStructuromeDB^20^. To allow for easy searching of transcripts, they are accessible by their ENST IDs and can be visualized within the database’s implementation of the Integrative Genomics Viewer (IGV) or downloaded as tracks for local analyses.

In this publication we show examples of how to access and use the RNAStructuromeDB to find transcripts of interest, and provide an example, using the *MAT2A* transcript (ENST00000306434.8), of how to use ScanFold data to develop structure–function hypotheses and identify regions for additional analyses. In doing this, we hope to lower the barrier of entry for researchers interested in studying any human mRNA transcript of interest by providing high quality RNA secondary structural models with a focus on regions of potential functional propensity.

## Results

### ScanFold-Scan of the human protein-coding transcriptome

**Figure 1:**
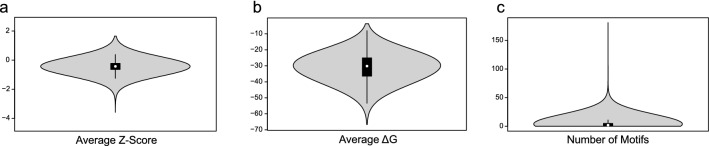
Violin plots of various average ScanFold metrics across the transcriptome. (**a**) The average windowed z-score that is shifted to a slightly negative overall value of −0.43 with outliers of −3.59 and +1.65. (**b**) The average windowed MFE (ΔG) that is centered around −31 kcal/mol with outliers of −66.68 kcal/mol and −3.68 kcal/mol. (**c**) The average number of motifs per transcript with z-score ≤ −2. The average was 3.92 and ranged from 0 to 181.

**Figure 2:**
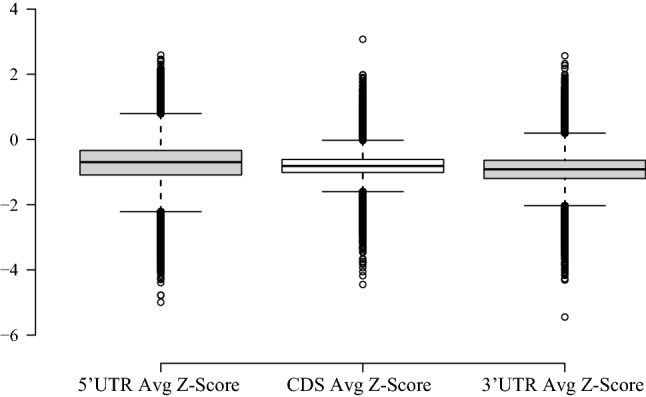
Box and whisker plot of the regional average per nucleotide z-score analysis across the transcriptome. The plot shows an overall decrease from the 5′UTR, to the CDS, to the 3′UTR with values of −0.71, −0.82, and −0.92, respectively.

ScanFold-Scan was applied to 100,552 transcripts isoforms associated with 20,342 protein-coding genes annotated in the human transcriptome (GENCODE)^35^; 188 transcripts were shorter than the window size used and were not analyzed (File S1). ScanFold-Scan generated several structural metrics for all analyzed transcripts including the MFE (ΔG of folding calculated by RNAfold), z-score (measure of ordered stability where each negative unit is a standard deviation more stable than random), and ED (measure of structural diversity in the ensemble of conformations). Average values were calculated (on a per transcript basis) for each metric and the percentage of windows with evidence of ordered stability (i.e., z-scores ≤ −1 and −2) were also tabulated (Table S1). Summaries of the windowed average z-score, MFE, and number of motifs per transcript can be visualized in Fig. 1a–c, respectively (all metrics generated can be found in File S2). Across the entire transcriptome the average windowed MFE was −31.00 kcal/mol, ranging from −66.68 kcal/mol (for ENST00000543234.1 or *INPPL1*) to −3.68 kcal/mol (for ENST00000361851.1 or *MT-ATP8*). The average windowed z-score was calculated to be −0.43, ranging from −3.59 (for ENST00000641394.1 or *SCYGR2*) to +1.65 (for ENST00000639391.1 or *RUVBL2*). In total, 4.95% of transcripts had an overall average windowed z-score ≤ −1 (one standard deviation more stable than random), indicating evidence of global ordered RNA structure. However, even for RNAs with overall positive z-scores, local regions were still predicted to have negative values, indicating that ordered structure likely plays roles across the transcriptome but to varying degrees. The z-score metric, in per nucleotide context, was also broken down by region (5′UTR, CDS, and 3′UTR) (Fig. 2), which shows a decrease in the overall average z-score (increased ordered stability) from the 5′UTR, to the CDS, to the 3′UTR. All data, broken down by location within the mRNAs of individual transcripts, can be found in the File S3, and the overall averages can be found in Table S1.

### Unusually ordered structural motifs are predicted throughout the protein coding transcriptome

The ScanFold-Fold analysis of z-scores and secondary structure models generated in the transcriptome-wide scans, resulted in unique (z-score) weighted consensus secondary structures being predicted across all transcripts. These consensus structures are formed from base pairs that reoccur across low z-score analysis windows, and they are biased towards ordered stability and likely functionality. Here, competition between potential pairing partners is considered, and a coverage-normalized z-score is used to more heavily weight base pair arrangements which consistently appear in low z-score windows (see “Methods”)^23,24^. The appearance of low z-score motifs varied in frequency across individual transcripts, but the total number of structures predicted across the transcriptome was 3,600,008. With z-score filters for structures ≤ −1 or −2, these numbers were reduced to 1,705,344 and 277,257, respectively. All transcriptome-wide data for individual transcripts can be found in File S2, and the overall averages can be found in Table S1. Additionally, all ScanFold-Scan and ScanFold-Fold data have been incorporated into the RNAStructuromeDB. Examples of global analyses of the data are below, followed by targeted examples of how to acquire, visualize, and generate structural hypotheses.

### Comparison of ScanFold predicted structures to Rfam human cis-regulatory elements

To demonstrate the utility of ScanFold at predicting known functional structures, we compared all predicted structures with a z-score ≤ −1 to the Rfam covariation models using the cmscan function in Infernal^36^. This comparison identified 25 of the 51 human cis-regulatory elements in the Rfam database, including internal ribosomal entry sites (IRESs), frame shifting elements (FSEs), RNA editing elements, 3′UTR stem loops, 5′UTR regulatory elements, iron response elements (IREs), selenocysteine insertion sequences (SECISs), and a prion pseudoknot (File S3). When comparing the predicted structures to the Rfam consensus model, we found that ScanFold recapitulated either the entire consensus model or major components of it. Unsurprisingly, we did not find many exact matches to the consensus models other than those of *MAT2A*, which will be discussed in detail later, as these models consider sequence and structure across many different species. In addition to identifying human cis-regulatory elements predicted by ScanFold we also determined the z-scores of each human cis-regulatory element, excluding those containing pseudoknots, found in Rfam. Of the 51 structures, 46 did not contain pseudoknots and were used to determine the median and average z-score of −0.92 and −1.21, respectively (File S4).

### Comparison of ScanFold data across differentially expressed genes

To assess if any trends could be elucidated within genes that are differentially expressed in specific tissues, additional analyses were completed. Here we analyzed the average windowed MFE (ΔG) and z-score for genes that exhibit tissue specific expression, genes that exhibit tissue enriched expression in at least one analyzed tissue, housekeeping genes (HKGs), and genes of transcription factors (TFs). We analyzed three subsets of tissue specific genes: (i) tissue enriched genes (at least four-fold higher mRNA level in a particular tissue compared to any other tissue); (ii) group enriched genes (at least four-fold higher average mRNA level in a group of 2–5 tissues compared to any other tissue); and (iii) enhanced genes (at least four-fold higher mRNA level in a particular tissue compared to the average level in all other tissues)^37,38^. The same analysis was also performed on subsets of specificity-based genes using their tissue distribution. These subsets contain expression in a single tissue, some tissue (more than one but less than one third of tissues), many tissues (at least one third of tissues), and all tissues (i.e., another iteration of HKGs)^37,38^. All expression dataset groups, number of genes in the group, number of genes analyzed, and definitions can be found in Table S2; and the results of these analyses can be found in Table 1 and File S2.

**Table 1:**
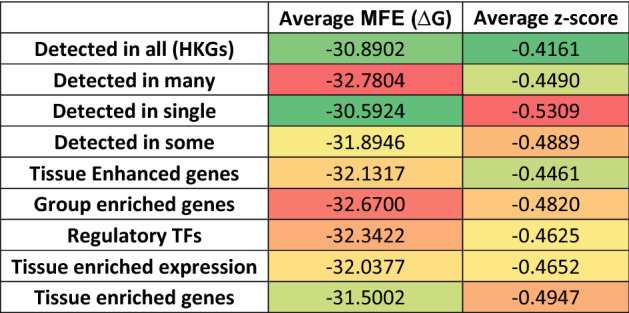
Analysis of average windowed MFE and z-score for differentially expressed genes in the human transcriptome.

The largest variation in average windowed MFE was found between transcripts detected in many tissues vs those detected in single tissue types, with that difference being 2.19 kcal/mol in favor of those detected in many tissues. Analysis of the average windowed z-scores revealed differences between transcripts “detected in all tissues (HKGs)” vs “detected in a single tissue” datasets. Transcripts detected in all tissues (HKGs) have the highest average z-scores at −0.416 whereas transcripts detected in a single tissue have the lowest average at −0.531. No other clear trends in average z-score were seen between any of the other datasets, and all data can be found in Table 1 and File S2.

Using these same datasets, we analyzed the regional (i.e., 5′UTR, CDS, and 3′UTR) per nucleotide average z-score data from ScanFold (Table 2 and File S5). When looking at these results across different expression datasets, a steady decrease in z-score from the 5′UTR to the 3′UTR is seen across all but one dataset—“detected in a single tissue”. In this case the CDS has the lowest z-scores, with the 5′UTR and 3′UTR only differing by a slight increase. Another thing to note from this analysis is, similar to what is seen for the average windowed values across these datasets, the transcripts detected in all tissues (HKGs) had the highest z-scores in all regions and transcripts detected in a single tissue had the lowest z-scores in all regions. All data can be found in Table 2 and File S5.

**Table 2:**
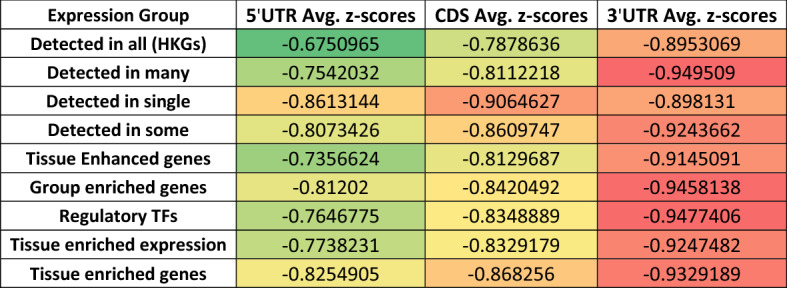
Analysis of average per nucleotide z-score across the 5′UTR, CDS, and 3′UTR of differentially expressed genes in the human transcriptome.

### Example data visualization and acquisition for MAT2A on RNAStructuromeDB

**Figure 3:**
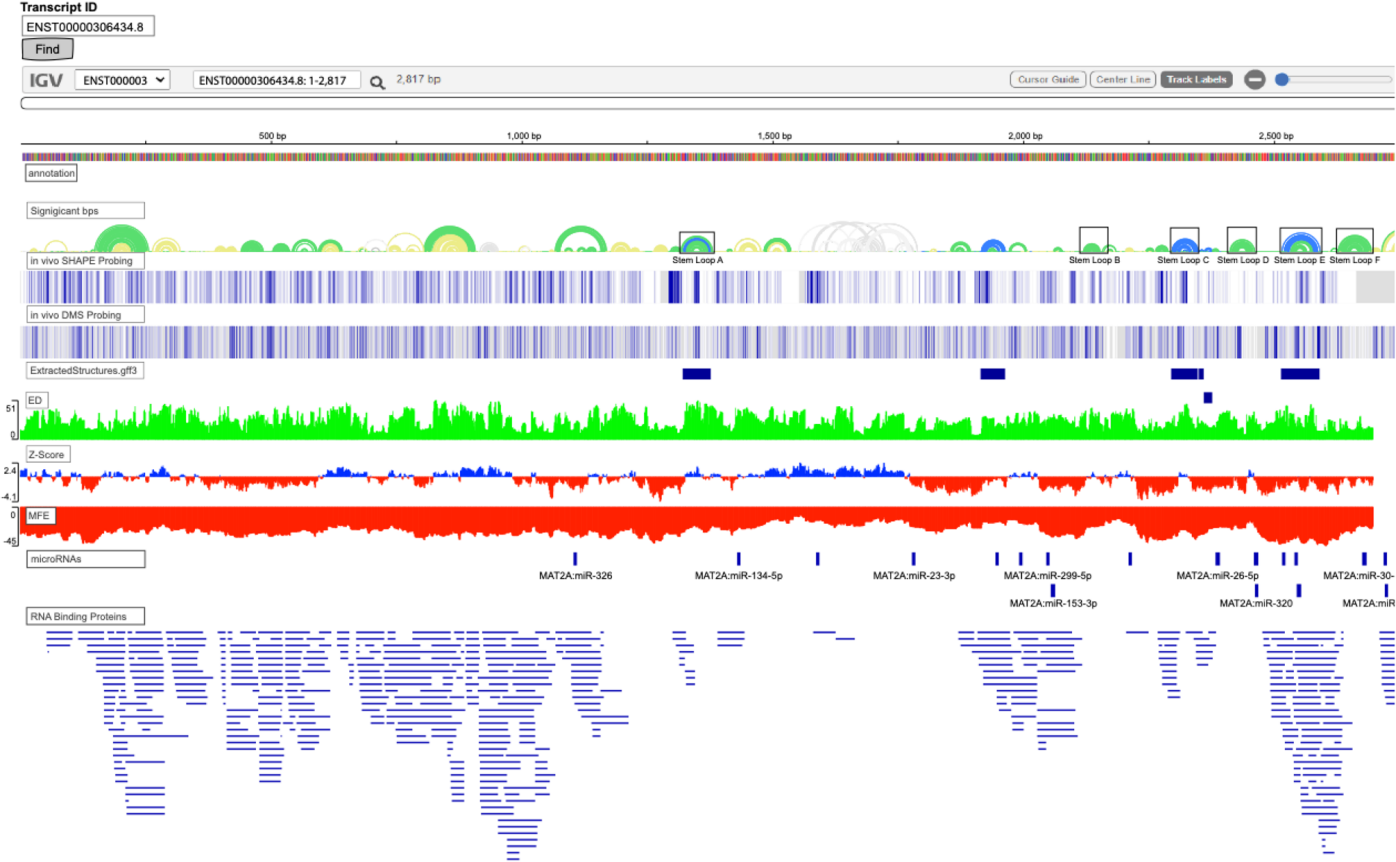
Example of the *MAT2A* transcript (ENST00000306434.8) data populated in the updated RNAStructuromeDB IGV window. From top to bottom the tracks have been organized into the annotation or sequence, significant bps or arc diagram, extracted structures with z-scores ≤ −2, ensemble diversity (ED), z-score, and MFE or ΔG. Additional in vivo DMS and SHAPE biochemical probing data (displayed as a heat map), microRNA sites, and RNA binding protein sites were generated and added to the window after ScanFold data acquisition. All track colors except significant bps were changed from their default color of gray to green for ED, blue for positive z-score, red for negative z-score, and red for MFE. The Rfam stem loop A-F structures of the 3′UTR have been annotated by boxed regions for ease of viewing.

To demonstrate how to use the RNAStructuromeDB data, we used *MAT2A* as an example. *MAT2A* encodes the protein Methionine adenosyl transferase 2A that catalyzes the reaction of L-methionine and ATP to S-adenosylmethionine (SAM), an essential methyl group donor^39–41^. To obtain the data, the RNAStructuromeDB was accessed (https://structurome.bb.iastate.edu/transcript-search), the ENST ID (ENST00000306434.8) was entered in the box on the left side above the IGV window, and the “find” button was selected. This searched all transcriptome-wide ScanFold data and populated the IGV window with all tracks including the sequence, secondary structure arc diagram, extracted structures of z-score ≤ −2, ED, MFE, and z-score. All populated data tracks were altered using the gear to the right of each track. All track colors were adjusted to show negative z-score and MFE values in red, positive z-score values in blue, and ED values in green. In Fig. 3, the IGV window with color changes and additional “omics” data (see “Discussion”) can be seen. In this example, six conserved structures were identified and annotated (Stem Loops A–F) based on data from Rfam^42,43^. These structures will be used to demonstrate how ScanFold data can model RNA secondary structure, highlight regions of likely functionality, and help generate structure function hypotheses that can be followed by experimental analyses.

### Utilizing ScanFold data for modeling RNA and hypothesis generation

ScanFold analysis of the *MAT2A* transcript identified thirty different structures with z-scores ≤ −1, of which six had z-scores ≤ −2. The entire transcript is less thermodynamically stable than the “typical” transcript with an average windowed MFE of − 27.27 kcal/mol. The MFE of the 5′UTR and CDS are consistent with only slight increases and decreases throughout. The 3′UTR does display large increases in MFE for regions that are predicted to be unstructured as well as between the cluster of stem loop structures near the 3′ most end of the transcript. The unstructured region may indicate sequence that is necessary for binding of regulatory trans-acting factors; whereas the other fluctuations seen in the 3′UTR indicate that there is potential to form stable structures across the transcript (relatively low MFE values), with some regions having the capability of forming uniquely stable and likely functional structures (indicated by low z-scores).

Of these thirty structures with unusual sequence-ordered stability, twenty-one were identified in the 3′UTR, while the remaining nine were identified in the CDS (Fig. 3). The relatively short 5′UTR (120 nt) had no significant structures predicted and had an average per nucleotide z-score of −0.40. All predicted CDS structures had a z-score ≤ −1, but none had a z-score ≤ −2. All predicted 3'UTR structures had a z-score ≤ −1, and six had a z-score ≤ −2. Of these six exceptionally stable motifs, three were previously described stem loop structures (archived in Rfam^42,43^) and three were novel motifs—a novel hairpin identified between stem loops A and B, and two short hairpins identified between stem loops C and D. These structures (stem loops A–E) are known cis-regulatory elements that act as recognition sites for *METTL16* m6A modifications^44,45^. The known structures identified by ScanFold show some of the lowest z-scores in the entire transcript, highlighting its ability to find functional RNA secondary structures. All *MAT2A* metrics mentioned above can be found in (Table S3).

**Figure 4:**
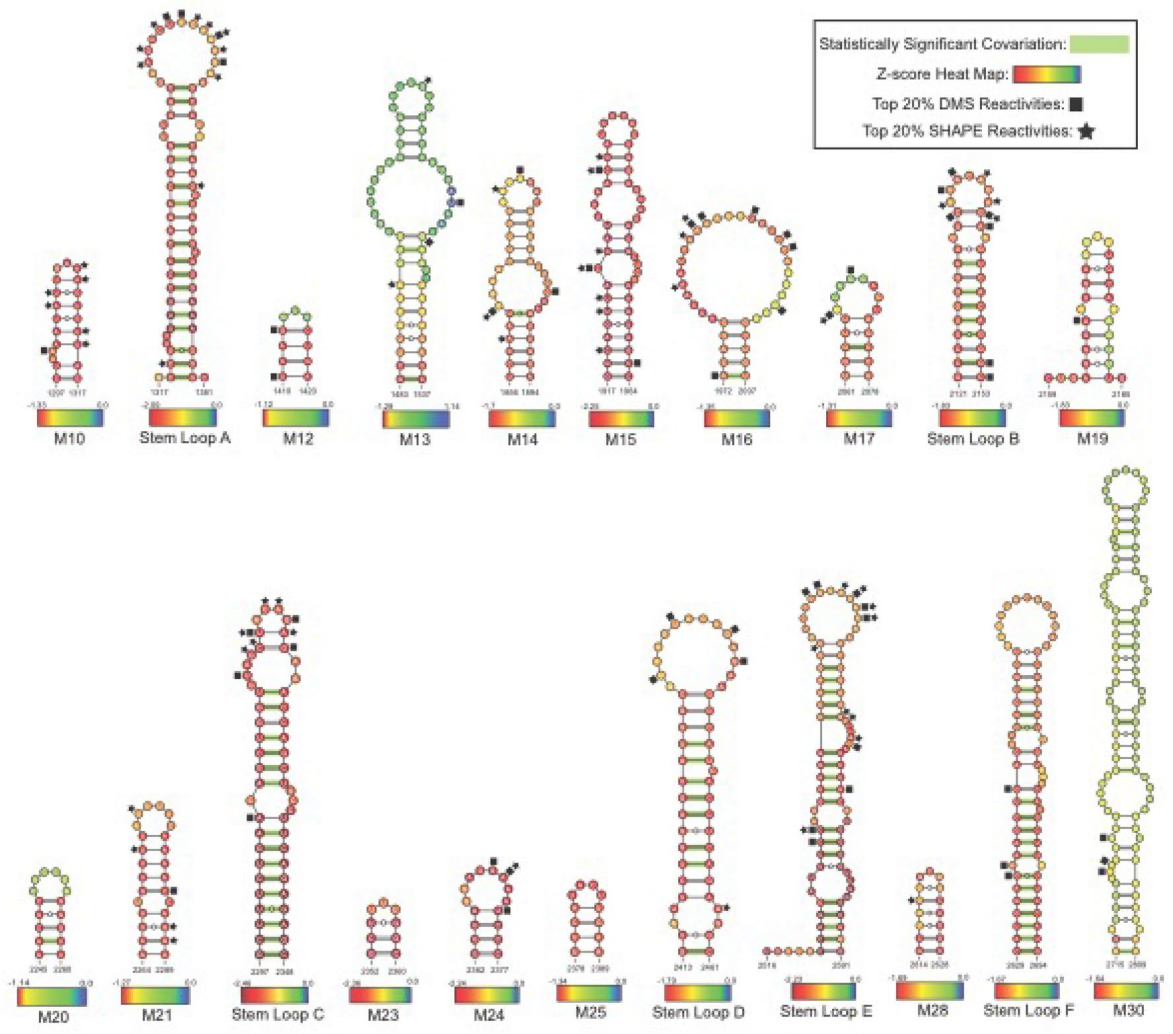
ScanFold predicted structural models of the *MAT2A* 3′UTR. All novel structures are annotated as M# (Motif #) and all known structures are annotated as in the Rfam database (Stem Loop A–E). Each nucleotide of these structures has been annotated with the per nucleotide z-score from the ScanFold final partners file, with red indicating the lowest z-scores (typically ≤ −2), yellow indicating z-scores ≤ −1, blue indicating z-score of 0, and combinations of these colors indicating z-scores that are in between −2, −1, and 0. All base pairs with statistically significant covariation have been annotated with green bars, and the top 20% of in vivo DMS and SHAPE reactivities have been annotated by squares and stars respectively.

Using all predicted structures with z-score ≤ −1, covariation analysis was performed using cm-builder^46,47^ (details in “Methods” section). Covariation is used as an additional layer of data that can suggest the potential for a structure–function relationship due to conservation of secondary structure through compensatory mutations across homologous gene sequences^48,49^. Covariation analysis of *MAT2A* found one structure in the CDS with significant covariation. Within the 3'UTR, however, twelve of twenty-one identified structures demonstrated some level of covariation, and stem loops A-E showed the highest levels of covariation. Stem loops A, C, D, E, and F contained between eight and seventeen covarying pairs with a power greater than 0.25 (or 25%), and B contained five covarying pairs with a power greater than 0.25 (or 25%) (Fig. 4). These results further indicate the ability of ScanFold to find potentially functional structures that are supported by many lines of evidence. All input files, Stockholm alignments, R-Scape/CaCoFold results, covariation models, and power analysis data can be found in File S6.

Using publicly available DMS and SHAPE biochemical probing data from the RASP database^50^, we were able to find additional support for our predicted structures. An ROC analysis comparing three DMS^51,52^ and three SHAPE^53^ datasets to the −1 and −2 z-score structure of *MAT2A*, revealed moderate support for the predicted structures. For DMS data, the AUC values ranged from 0.58 to 0.64 for all structures with a z-scores ≤ −1 and from 0.59 to 0.66 for all structures with a z-scores ≤ −2. For SHAPE data, the AUC values ranged from 0.52 to 0.54 for all structures with a z-scores ≤ −1 and from 0.51 to 0.58 for all structures with a z-scores ≤ −2. When the top 20% of reactivity values^54^ are overlaid (not used as constraints) on all −1 and −2 z-score structure in the 3′UTR of *MAT2A*, the reactivities map predominantly to single stranded regions (Fig. 4), offering experimental support for the predicted structures. All ROC analysis data can be found in File S7.

## Discussion

### Transcriptome-wide analysis

ScanFold-Scan data for the 100,552 annotated transcripts isoforms from 20,342 human protein-coding genes revealed variation in metrics across transcripts. For example, the lowest and highest average windowed z-scores are −3.59 (ENST00000641394.1 or SCYGR2) and +1.65 (ENST00000639391.1 or RUVBL2) respectively, and the lowest and highest average windowed MFEs are −66.68 kcal/mol (ENST00000543234.1 or INPPL1) and −3.59 kcal/mol (ENST00000361851.1 or MT-ATP8), respectively. These variations indicate how different transcripts can form more or less structure with potential for function (Table S1, Fig. 1, and File S2). An additionally observed trend is the decreasing z-score from the 5′UTR to the 3'UTR (Table S1, Fig. 2, and File S5). This decrease in z-score is associated with an increased propensity for unusually stable and potentially functional structures towards the 3′UTR. Across all transcripts, the 5'UTR is less populated with significant structure than the CDS, which contains less significant evidence of ordered structure than the 3'UTR. This supports the body of work that finds the presence of highly stable RNA structure in the 5'UTR to generally be inhibitory to efficient ribosomal scanning^4,32^. For the CDS, there are some cases where more potential for uniquely stable structure may be ideal to slow the ribosome, allow the protein time to fold correctly, and allow binding of necessary trans-acting factors^4,32^. Similar to other studies, we also noted the most potential for stable and likely functional structure is within 3′UTRs—regions rich in cis- and trans-regulatory elements that may utilize local secondary structure in the regulation of expression^4,32,33^. Notably, our results suggest that *specifically* ordered (low z-score) structure could be playing roles in these processes (vs simply RNA secondary structural stability). Additionally, our comparison of Rfam human cis-regulatory elements to ScanFold predicted structures indicates that ScanFold can find known functional elements as well as novel, potentially functional structures. We also find that the use of a −1 z-score cutoff is appropriate for finding these types of structures, as the average z-score of the human cis-regulatory elements in Rfam is −1.21 and their average length is less than 150 nt, which aligns well with the 120 nt window used here. Notably, even when regulatory elements span >120 nt, shorter fragments of structure can still be identified—drawing attention to sites that can be further analyzed for longer-range structure (e.g., as we did for *MAT2A*).

### ScanFold data for differentially expressed genes

The differential expression datasets were compared against ScanFold results (Table 1). The lowest and highest average windowed MFEs from “detected in all tissues” are −63.19 kcal/mol (ENST00000511587.1 or ZBED3) and −3.67 kcal/mol (ENST00000361851.1 or MT-ATP8), respectively, and the lowest and highest MFEs from “detected in single tissue” are −57.97 kcal/mol (ENST00000434396.1 or ANKRD63) and −13.61 kcal/mol (ENST00000487798.5 or CYLC2), respectively. The MFE results indicate that all analyzed transcripts, regardless of their expression profiles, have similar predicted thermodynamic stability^26,55,56^. Although the general stability of all transcripts was relatively constant, the levels of *uniquely* stable and potentially functional structures varied.

The results from the comparison of average windowed z-scores among the expression datasets demonstrated that the z-scores of transcripts detected in all tissues (HKGs) are higher than those of transcripts detected in a single tissue (Table 1). We find that the lowest and highest average z-scores from “detected in all tissues” are −2.89 (ENST00000527353.1 or PIGY) and +1.49 (ENST00000628044.1 or PFKL), respectively, and the lowest and highest z-scores from “detected in a single tissue” are −1.89 (ENST00000390348.2 or TRGV1) and +1.12 (ENST00000518835.1 or ODF1), respectively. These results coincide with the idea that lower z-scores indicate more significantly stable and potentially functional structure that could play a role in regulating the expression of transcripts that are differentially expressed, especially those found in single a tissue. (Table 1 and File S2). Transcripts with the most restricted tissue expression may have a need for more regulation of expression that coincides with more significantly stable secondary structure (lower z-scores) that can stabilize transcripts, leading to longer half-lives, and increased levels of mRNA^4,5,57^.

Comparison of the regional per nucleotide average z-score data to the gene expression datasets shows a similar trend to that of the entire transcriptome where all groups, except those detected in a single tissue, displayed a decrease in z-score from the 5′UTR to the 3′UTR (Table 2 and File S5). This trend is expected as higher z-scores are indicative of less significantly stable and likely functional structures^23,24^. This decrease across transcripts is likely because the 3′UTR has a greater capacity for containing evolved functional structure as compared to the 5′UTR and CDS^4,58,59^. The 5′UTR may require some locally ordered structure to mediate interactions with regulatory factors, but an excess of structure (especially significantly stable structure) can begin to inhibit ribosomal scanning and translation^4,60,61^. The coding sequence has potential to contain regulatory structures but is under additional constraints (as opposed to the 3′UTR) to maintain codon sequence order^4,8^. Contrary to the 5′UTR and CDS, the 3′UTR potentially has fewer restrictions on its length and sequence composition, allowing it to contain many cis-regulatory elements^58,59^. It is therefore intuitive that there will be lower z-scores and more significantly stable, potentially functional structures in the 3′UTR for regulation of expression and recruitment of trans-acting factors^4,7,8,62^. Interestingly, in the case of transcripts detected in single tissue, the average regional z-scores are lowest in the CDS by a slight margin (Table 2 and File S5). This may be indicative of the need to finely tune the levels of these transcripts to maintain proper function in these tissue types^4,8,63,64^. The higher z-scores found across all regions of transcripts detected in all tissues (HKGs) could be evidence that these transcripts do not need to be regulated as tightly as others and therefore form fewer uniquely stable and potentially regulatory structures.

### ***MAT2A ***3′UTR stem loop structures

The analysis of the *MAT2A* transcript illustrates the ability of ScanFold to find and fold potentially functional and druggable RNA secondary structures, as well as guide hypothesis generation. *MAT2A* was previously found to contain six conserved hairpins or stem loop structures across the 3′UTR that are annotated as cis-regulatory elements^42,43^. These cis-regulatory elements were found to be involved in regulation of SAM levels through methylation of these hairpins by *METTL16*, which promotes efficient posttranscriptional and/or co-transcriptional splicing^44,45,65^. This process works through a feedback loop of SAM levels. When SAM levels are high, the hairpins are not methylated due to autoinhibition of *METTL16* and both splicing of *MAT2A* and levels of SAM biosynthesis are limited. When SAM levels are low, *METTL16* is activated, the hairpins are methylated, and both efficient splicing of *MAT2A* and increased translation of the transferase responsible for biosynthesis of SAM occurs^30,44,45,66^.

Stem loops A, C, and D are validated structural motifs, finding initial support from in-line probing experiments^42,65^; notably, all hairpins were almost perfectly predicted by ScanFold. The minor differences in predictions compared to Rfam consensus structures are the formation of a bulge in the basal stem rather than an internal loop (A), pairing of two nucleotides that decrease the size of the terminal loop (C), and the formation of a longer stem containing a large bulge near the base (D) (Fig. 4). The subtle differences could be attributed to “breathing” of loops and bulges during probing experiments, native structural dynamics that are not seen in predictions due to the occurrence of low z-score paired nucleotides across the analysis windows that out-compete any other potential conformations, or slight deviations from the Rfam consensus structure that accounts for sequence and structure across multiple species. The structures of stem loops B, E, and F have not been validated by in line probing, but ScanFold does predict the top half of stem loop B and all of stem loop E and F that match the structures archived in Rfam^42,43^. Additionally, structures A and F have been crystalized in complex with *METTL16*, and were shown to have functional significance^30^, highlighting ScanFold’s ability to predict potentially functional RNA secondary structures that can be experimentally validated^44,45^. DMS and SHAPE probing data provide evidence for ScanFold structure models. An ROC analysis of both −1 and −2 z-score structures showed the most support from one in vivo DMS dataset^51^ with AUC values of 0.64 and 0.66, respectively (File S7). In the case of SHAPE data, the −1 and −2 z-score structures showed less support with the best AUC values from one in vivo SHAPE dataset^52^ being 0.54 and 0.57, respectively (File S7). When the top 20% of DMS and SHAPE reactivities^54^ are overlaid on the predicted structural models it can be seen that both known and novel structures are generally consistent (Figs. 3 and 4) with the majority of apparent inconsistencies appearing adjacent to single stranded regions.

With respect to the novel predicted motifs in *MAT2A*, the results of cm-builder suggest functionality; as alongside the known hairpins (Fig. 4), eleven out of thirty ScanFold identified motifs show moderate to high levels of statistically significant covariation while two others show low levels of statistically significant covariation (File S6 and Fig. 4). Their preservation across many species and the observation of compensatory structure-preserving mutations offers confirmatory evidence of the model structure and potential functionality. The novel regions of *MAT2A* merit further investigation to assess their potential functionality. The brief section below outlines how structure/function hypotheses could be framed by combining ScanFold data with other “omics” data.

In addition to ScanFold predictions and covariation data there are other lines of evidence for potential function. This comes in the form of annotation data or publicly available “omics” datasets, which help guide researchers to home in on regions of genes that have propensity to be involved in different interactions that could mediate their function. Using the genomic coordinates of *MAT2A*, we were able to search several datasets including eCLIP RBP data^67,68^, OregAnno regulatory data^69^, mRNA m6A modification data^70^, SNP mutational data^71,72^, PolyA site data^73^, RefSeq functional elements^74^, repeat elements^75^, and microRNA site data^76,77^ to find information for facilitating generation of functional hypotheses. Of these data sources, only microRNA sites and RNA binding protein sites (RBPs) overlapped *MAT2A*. There are multiple microRNA binding sites located within stem loops D–F as well as many of the novel structures found across the 3′UTR that could provide additional regulation of this transcript (Fig. 3). The transcript was also found to potentially be bound by a variety of RBPs; many of which overlap novel structural elements predicted throughout the 5′UTR, CDS, and 3′UTR (Fig. 3). Many of these RBPs including FUS, PABPN1, and TIA1 are known regulators of transcription, translation, RNA splicing, RNA transport, polyadenylation, and other important cellular processes. This combination of both ScanFold and annotation data can be applied to any target of interest to provide valuable insights into the potential functional roles of predicted structures, allowing researchers to strategically design experiments to test their hypotheses. Notably, motifs with strong evidence of ordered stability and functionality are not only candidates for additional basic research (e.g., structural biology to characterize 3D structure or assays of function), but also represent ideal targets for RNA-binding drugs. To facilitate such work, all annotation data and protocols on how to extract the desired data are available in a filtered (RNA centric) and downloadable format on the RNAstructuromeDB^78^.

## Conclusions

The data generated in this study provides a great entry point for researchers interested in studying RNA secondary structure at any level. Depositing this data on the updated RNAStructuromeDB makes acquiring and visualizing structural information for any transcript of interest an easy task. The *MAT2A* example demonstrated the utility and dependability of ScanFold predictions to find potentially functional and druggable RNA secondary structures that are both known (experimentally validated via probing and structural biology techniques) and novel. Using these prediction data alongside covariation and annotation data, valuable insights can be gleaned and many new hypotheses for further experimentation can be developed. With these new transcriptome data available, they can be used in tandem with the previous data from the entire human genome, making the RNAStructuromeDB a useful resource to access a wide variety RNA secondary structural information. We hope that this resource can help drive the fields of basic RNA research and RNA therapeutics forward by lowering the barrier of entry for researchers interested in studying any human mRNA transcript of interest.

## Methods

### Transcript fasta data acquisition

All transcriptome data was acquired from the GENCODE database Release 33 (GRCh38.p13)^35^. A single fasta containing all protein-coding transcript sequences was downloaded and used as input for ScanFold (File S8).

### ScanFold

ScanFold is an RNA sequence scanning pipeline which attempts to identify uniquely stable and potentially functional RNA secondary structures. In brief, ScanFold is composed of a scanning step and a folding step. In ScanFold-Scan, a scanning window analysis of the entire sequence is performed. The sequence of each window is folded via RNAfold 2.0^21^ to calculate its native MFE and associated base pairing. That sequence is then shuffled using mononucleotide or dinucleotide shuffling to produce a user defined number of random sequences. Each of the randomized sequences is then folded to calculate an average MFE value for use in the calculation of the thermodynamic *z*-score. After the scanning step is complete, ScanFold-Fold analyzes the z-score calculations to generate consensus secondary structures across the sequence based on paired nucleotides that reoccur across low z-score analysis windows^23^. To find the best pairing partners, all competing pairs are analyzed using the Z_norm_ metric, which accounts for all predicted interactions of each nucleotide^24^. To do this, another metric, the Z_sum_, is calculated, where all occurrences of a nucleotide base pairing are recorded and summed. This Z_sum_ value is then divided by the number of windows the nucleotide is paired in to provide the Z_norm_^24^. This gives a coverage-normalized z-score that more heavily weights base pair arrangements which consistently appear in low z-score windows, providing a normalized metric for comparison of regions with lower window coverage (near the ends, where nucleotides are covered by only a few windows). The pairing that has the lowest Z_norm_ is then selected as the most favorable arrangement and used to create the consensus structural model^23,24^. These structures are biased towards ordered stability and likely functionality. All structures having at least one base pair with z-score ≤ −2 are then extracted for use in further downstream analyses.

Metrics obtained from ScanFold include the MFE or ΔG (a measure of thermodynamic stability), z-score (a measure of ordered stability that can indicate potential function), ensemble diversity (ED; a measure of predicted structure’s conformational volatility), and a p-value (a quality control metric)^23^. The MFE is estimated by the predicted Gibb’s folding free energy change (the ΔG°) going from a fully denatured (random coil) RNA to an ordered 2D structure, where more negative values indicate greater stability^23,24^. The z-scores are used to identify structures that have propensity for ordered stability, where negative values indicate the number of standard deviations more stable the native sequence is compared to any randomized sequence version^22,23^. The ED uses the RNA partition function to compare the structural distance between all Boltzmann-weighted conformations^27,28,79^. Lower ED values indicate a single dominant conformation, while higher EDs suggest multiple conformations or a lack of defined structure^79,80^. The arc diagrams depict the weighted z-score structures where blue, green, and yellow arcs indicate z-scores ≤ −2, ≤ −1, and < 0, respectively. For more information on the program, its output files, and their significance see the original ScanFold paper and methods paper^23,24^.

In our analysis of all protein coding transcripts, the following parameters were used: a 120 nt window size, a 1 nt step size, 100 randomizations per window, mononucleotide shuffling, 37 °C temperature, competition of 1 (to demand that only one unique base pair per nucleotide is possible), and extraction of structures with z-score ≤ −2. During our analyses we found 188 annotated transcripts that were too short to be scanned using the 120nt window size (i.e., transcript length was under 120 nt). For these short transcripts, no ScanFold data was produced (File S1).

### Comparison of ScanFold structures to Rfam human cis-regulatory elements

The Rfam.cm (version 14.8)^42^ covariation model file was downloaded and used to compare against the sequences of all ScanFold structures with a z-score ≤ −1 using Infernal^36^. The Rfam.cm file was unzipped and the cmpress command was run. The cmscan command was then run as follows: cmscan -rfam -cut_ga -nohmmonly -tblout transcriptome.tblout -fmt 2\transcriptome.fa > transcriptome.cmscan. After completion of the cmscan step the tblout file was analyzed in Excel to find ScanFold structures that matched known human cis-regulatory elements in the Rfam database. The results of the cmscan run can be found in File S3. To find the z-scores of all human cis-regulatory elements that do not contain pseudoknots, each sequence was downloaded from Rfam and the script “HTP_dG_ZScore.pl” (https://github.com/moss-lab/Transcriptome_Scripts) was ran to calculate the MFE, z-score, and p-value for each sequence. For consistency with all other transcriptome data, 100 randomizations were used to calculate the z-scores for each sequence. The results of this analysis can be found in File S4.

### ROC analysis

The ROC analysis was performed on ScanFold -1 and -2 z-score predictions following a previously establish protocol^29^. Briefly, reactivity value thresholds were sequentially set from lowest to highest value at 1% intervals (i.e. 0–100% constrained) for three DMS^51,52^ and three SHAPE^53^ reactivity datasets from the RASP database^50^. The −1 and −2 z-score CT files from ScanFold were cross referenced to these reactivity datasets and used to find the true positive rate (TPR) and false positive rates (FPR) for each comparison. In this analysis, the TPR and FPR are represented by Eqs. (1) and (2) below:

1 $$\begin{array}{c}TPR=\frac{TP}{\left(TP+FN\right)}\end{array}$$

2 $$\begin{array}{c}FPR=\frac{FP}{\left(FP+TN\right)}\end{array}$$

The true positive (TP) is defined as being *paired* in the given CT file and *paired* at the defined reactivity threshold, the false negative (FN) is *paired* in the CT file and *unpaired* at the reactivity threshold. The false positive (FP) is *unpaired* in the CT file and *paired* at the reactivity threshold, and the true negative (TN) is *unpaired* in the CT file and *unpaired* at the given reactivity threshold. When the threshold is set to 0%, TPR and FPR will be equal to zero, and when the reactivity threshold is set to 100%, TPR and FPR will be equal to one. If a given RNA secondary structure model is truly random, when compared to increasing reactivity thresholds from a probing data set, then the TPR and FPR should increase proportionately yielding a linear trend in the plot. However, if the RNA secondary structure model agrees with the reactivity data set, the TPR should initially rise faster than the FPR, creating a larger area under the curve (AUC) and producing a curve on the plot. In this way, we can quantitatively assess and compare each model's ability to fit the data via their respective AUCs. All the ROC and AUC analysis can be found in File S7.

### Acquisition of expression data

All gene expression data was obtained from The Human Protein Atlas^37,38^ on February 1, 2022. These datasets contained genes that exhibit tissue specific expression, genes that exhibit tissue enriched expression in at least one analyzed tissue, housekeeping genes (HKGs), and genes of transcription factors (TFs). There are 10,992 genes that exhibit tissue specific expression, 8839 HKGs, and 1490 TF genes. Within the list of genes exhibiting tissue specific expression, there are subsets of tissue specific genes including tissue enriched genes (at least four-fold higher mRNA level in a particular tissue compared to any other tissue), group enriched genes (at least four-fold higher average mRNA level in a group of 2–5 tissues compared to any other tissue), and enhanced genes (at least four-fold higher mRNA level in a particular tissue compared to the average level in all other tissues)^37,38^. Within these subsets, there are 3107 tissue enhanced genes, 1691 group enriched genes, and 6194 enhanced genes. Additionally, we found subsets of specificity-based genes using their tissue distribution. These subsets contain detection in a single tissue, some tissue (more than one but less than one third of tissues), many tissues (at least one third of tissues), and all tissues (HKGs). Within these subsets, there are 1062 genes in a single tissue, 3368 genes in some tissues, 5956 genes in many tissues, and 8839 genes in all tissues or HKGs. All definitions of these specificities and distribution are based on the nomenclature used in the Human Protein Atlas^37,38^. All expression dataset groups, number of genes in each group, number of genes analyzed, and definitions can be found in Table S2.

### Updates to RNAStructuromeDB

Using the original RNAStructuromeDB^20^ interface for displaying ScanFold data, we added the embeddable IGV application^81^ into our database with an additional text field for controlling the files that are loaded. These updates were then added to the transcript search tab within the RNAStructuromeDB, allowing all transcript data to be searched for and displayed. This update now allows users to search for lower resolution (40 nt step size) genome wide data (Data Search or JBrowse tab) or higher resolution (1 nt step size) protein-coding transcriptome data (Transcript Search) all in a single, easy to use database.

### Guide to use the RNAStructuromeDB

To access and visualize data on RNAStructuromeDB^20^, the following link can be used (https://structurome.bb.iastate.edu/transcript-search) or the “transcript search” tab within the RNAStructuromeDB can be used. Once on the database, simply type the ENST ID of interest (with or without the version number) into the transcript ID search bar and click “find”. This will update the IGV window and display the transcript sequence, base pair track or arc diagram, extracted structures track (z-score ≤ −2), ED track, MFE track, and z-score track. Once all tracks are visible, they can be manipulated in any order by clicking the gray bar on the right side and dragging it up or down. The track heights, colors, and names can also be changed by clicking the gear to the right of each track, selecting the option of interest from the drop-down menu, and making the desired change. In the example provided here, the colors were adjusted to show negative z-score and MFE values in red, positive z-score values in blue, and ED values in green for easy differentiation of each.

### Covariation analysis of predicted structures

All thirty *MAT2A* structures with a z-score ≤ −1 were analyzed for covariation using the cm-builder Perl script^47^. The script builds off the RNAFramework toolkit^82^ and utilizes Infernal (release 1.1.2)^36^ to build and find covariance models for predicted ScanFold structures. The Infernal database was created using the NCBI RefSeq database in BLAST^83^. Using the transcript sequence scanned by ScanFold, the NCBI RefSeq database was searched using the following parameters: blastn, gap open 5, gap extend 2, reward 1, penalty − 1, max target sequences of 5000. All pseudogenes and “like” sequence were deselected and the resulting list was downloaded and used in following analyses. The resulting structural alignment files (in Stockholm format) were tested for covarying base pairs and analyzed with the CaCoFold algorithm using R-Scape v2^48^; statistical significance was evaluated by the APC corrected G-test^46,49^ using the default E value of 0.05. The power files generated were analyzed using an in-house script that breaks down the power of covarying base pairs into 0–0.1, 0.1–0.25, and ≥ 0.25 for determining base pairs with the best results. All input files, Stockholm alignments, R-Scape/CaCoFold results, and power analysis data can be found in File S6.

### Python scripts used in analyses

Several python scripts were written and used to analyze the large dataset generated from ScanFold (https://github.com/moss-lab/Transcriptome_Scripts). The script “transcriptome_metrics.py” was used on the ScanFold out file and the extracted structures gff3 file to parse out the average windowed ΔG, z-score, number of windows generated, percent of windows with a z-score ≤ − 1, percent of windows with a z-score ≤ − 2, sequence length, and number of motifs for all transcriptome wide data and expression data (File S2). The script “HTP_dG_ZScore.pl” was used to calculate the z-scores for human cis-regulatory elements in the Rfam database (File S4). The script “regional_zavg.py” was used on the z-avg wig file to find the regional average per nucleotide z-scores (i.e., 5′UTR, CDS, and 3′UTR) across the transcriptome wide dataset (File S5). The script “differential_expression_metrics.py” was used to parse the Human Protein Atlas expression datasets against the output from “transcriptome_metrics.py” (File S5). The script “cm_power_parser.py” was used to parse cm-builder power files and output power of covarying base pairs into binned groups of 0–0.1, 0.1–0.25, and ≥ 0.25 (File S6).

## Supplementary Information

Supplementary Information.

## Data Availability

All supplemental data is available online at Scientific Reports. All other data reported here can be found on the RNAStructuromeDB (https://structurome.bb.iastate.edu/transcript-search).
