# Supplementary material for: Expansion of the RNAStructuromeDB to include secondary structural data spanning the human protein-coding transcriptome
Source: Sci Rep. 2022 Aug 25;12:14515. doi: 10.1038/s41598-022-18699-3 (PMC9403969; doi:10.1038/s41598-022-18699-3)
Supplement: Supplementary file 1 — Supplementary Information. [file 41598_2022_18699_MOESM1_ESM.zip › Supplemental/File S1.docx]

ENST00000390530.1

ENST00000637437.1

ENST00000390512.2

ENST00000523680.1

ENST00000390511.1

ENST00000390496.1

ENST00000390418.1

ENST00000390339.1

ENST00000636628.1

ENST00000488476.1

ENST00000390240.2

ENST00000637492.1

ENST00000390239.2

ENST00000390486.1

ENST00000637820.1

ENST00000390412.1

ENST00000626298.1

ENST00000390333.1

ENST00000390520.1

ENST00000390330.2

ENST00000636994.1

ENST00000452198.1

ENST00000390521.1

ENST00000637307.1

ENST00000390413.1

ENST00000390487.1

ENST00000422426.5

ENST00000390238.2

ENST00000390560.2

ENST00000636812.1

ENST00000390419.1

ENST00000633713.1

ENST00000565533.1

ENST00000390497.1

ENST00000390584.1

ENST00000637097.1

ENST00000636968.1

ENST00000390479.2

ENST00000390510.1

ENST00000636840.1

ENST00000612375.1

ENST00000637873.1

ENST00000637204.1

ENST00000390569.1

ENST00000626828.1

ENST00000390531.1

ENST00000631166.1

ENST00000390533.1

ENST00000625892.1

ENST00000520028.1

ENST00000632684.1

ENST00000632041.1

ENST00000390495.1

ENST00000390508.1

ENST00000450276.1

ENST00000632951.1

ENST00000390518.1

ENST00000628529.2

ENST00000454908.1

ENST00000625905.1

ENST00000630530.1

ENST00000562187.1

ENST00000390485.1

ENST00000636483.1

ENST00000390502.1

ENST00000390523.1

ENST00000638614.1

ENST00000635865.1

ENST00000636337.1

ENST00000390503.1

ENST00000390484.1

ENST00000390328.2

ENST00000390473.1

ENST00000461719.1

ENST00000390519.1

ENST00000636902.1

ENST00000390242.2

ENST00000390509.1

ENST00000636257.1

ENST00000605284.1

ENST00000390494.1

ENST00000390528.1

ENST00000390338.2

ENST00000638016.1

ENST00000390513.1

ENST00000636406.1

ENST00000638037.1

ENST00000390532.1

ENST00000390326.2

ENST00000390535.2

ENST00000390517.1

ENST00000633780.1

ENST00000390490.1

ENST00000390583.1

ENST00000390574.1

ENST00000636916.1

ENST00000390414.1

ENST00000390507.1

ENST00000636201.1

ENST00000390241.3

ENST00000649155.1

ENST00000390526.1

ENST00000434667.3

ENST00000390527.1

ENST00000390588.1

ENST00000646142.1

ENST00000637188.1

ENST00000390415.1

ENST00000604838.1

ENST00000390506.1

ENST00000454691.1

ENST00000630143.1

ENST00000636221.1

ENST00000390334.1

ENST00000604446.1

ENST00000390476.1

ENST00000390565.1

ENST00000614481.1

ENST00000255006.11

ENST00000627077.1

ENST00000390575.1

ENST00000390491.1

ENST00000390516.1

ENST00000647139.1

ENST00000627400.1

ENST00000390327.2

ENST00000638032.1

ENST00000625535.1

ENST00000390489.1

ENST00000674075.1

ENST00000637026.1

ENST00000559547.1

ENST00000390536.2

ENST00000448914.1

ENST00000629101.1

ENST00000390514.1

ENST00000390493.1

ENST00000463911.1

ENST00000390580.1

ENST00000451044.1

ENST00000390564.2

ENST00000638959.1

ENST00000390480.2

ENST00000390590.1

ENST00000390483.1

ENST00000390474.1

ENST00000390567.1

ENST00000634000.1

ENST00000390417.1

ENST00000394143.6

ENST00000390504.1

ENST00000390499.1

ENST00000637867.1

ENST00000638078.1

ENST00000390525.1

ENST00000390524.1

ENST00000638314.1

ENST00000376954.5

ENST00000434970.2

ENST00000390498.1

ENST00000621643.1

ENST00000390416.1

ENST00000390505.1

ENST00000390337.1

ENST00000635842.1

ENST00000390475.1

ENST00000390482.1

ENST00000637082.1

ENST00000648400.1

ENST00000604642.1

ENST00000415118.1

ENST00000430425.1

ENST00000628959.1

ENST00000636645.1

ENST00000635831.1

ENST00000390492.1

ENST00000390581.1

ENST00000635336.1

ENST00000390515.1

ENST00000633553.1

ENST00000439842.1

ENST00000637134.1

ENST00000639611.1

ENST00000642841.1

ENST00000361446.5

ENST00000390534.1

ENST00000390488.1

ENST00000629769.2
