# Supplementary material for: Expansion of the RNAStructuromeDB to include secondary structural data spanning the human protein-coding transcriptome
Source: Sci Rep. 2022 Aug 25;12:14515. doi: 10.1038/s41598-022-18699-3 (PMC9403969; doi:10.1038/s41598-022-18699-3)
Supplement: Supplementary file 1 — Supplementary Information. [file 41598_2022_18699_MOESM1_ESM.zip › Supplemental/Supplemental_Descriptions.docx]

**File S1.** This file contains all ENST transcript IDs for those with sequences shorter than 120 nucleotides. This set of 188 IDs are the transcripts that were not scanned and analyzed.

**File S2.** This file contains individual and average ScanFold metrics (MFE, z-score, number of windows analyzed, number of windows with a z-score ≤ -1, percent of windows with a z-score ≤ -1, number of windows with a z-score ≤ -2, percent of windows with a z-score ≤ -2, sequence length, and number of motifs per transcript with a z-score ≤ -2) for all transcripts in the transcriptome-wide dataset. It also contains the same data for all transcripts in the differential expression datasets. Table 1 is also located within this file.

**File S3.** This file contains the matches between the human cis-regulatory elements in the Rfam.cm file and all -1 and -2 z-score structures in the protein-coding transcriptome predicted by ScanFold.

**File S4.** This file contains the MFE, z-score, and p-value calculated by the HTP_dG_ZScore.pl script for all human cis-regulatory elements in Rfam that do not contain a pseudoknot. A violin plot and its associated metrics can also be found here.

**File S5.** This file contains windowed individual z-score data for all transcripts in the transcriptome-wide dataset, and average per nucleotide regional (5’UTR, CDS, and 3’UTR) z-score, MFE, and ED data for all transcripts in the transcriptome-wide dataset. The length of these regions is also annotated for each transcript. It also contains the same data for all transcripts in the differential expression datasets.

**File S6.** This file contains all cm-builder covariation analysis data for all 30 structures with z-scores ≤ -1. This includes the R-Scape and CaCofold input Blast sequence, dot bracket sequence and structure files, output alignments and power files, intermediate files, covariance models, and an excel file for the analyzed power data.

**File S7.** This file contains all the data used in the ROC analysis. This includes the reactivity files, ct files, constraint files, scripts, probing information, and analyzed data plotted in excel.

**File S8.** This is the transcriptome fasta file containing all 100,552 transcripts analyzed in this study. This file was acquired from the GENCODE database Release 33 GRCh38.p13 on March 27, 2020.

**Table S1.** This file contains the average, highest, and lowest values for all transcriptome metrics; and the regional average z-scores for all transcriptome metrics.

**Table S2.** This table contains specifics on all expression datasets including the group name of each group, number of genes in each group, number of genes analyzed in this study, the Human Protein Atlas definition of each group.

**Table S3.** This file contains all major MAT2A ScanFold metrics for the entire transcript. It also contains the metrics from regional analysis as well as the ensemble diversity and z-score metrics for the stem loop structures found in the 3’UTR.
