# Supplementary material for: Expansion of the RNAStructuromeDB to include secondary structural data spanning the human protein-coding transcriptome
Source: Sci Rep. 2022 Aug 25;12:14515. doi: 10.1038/s41598-022-18699-3 (PMC9403969; doi:10.1038/s41598-022-18699-3)
Supplement: Supplementary file 1 — Supplementary Information. [file 41598_2022_18699_MOESM1_ESM.zip › Supplemental/Table S1.docx]

| **Calculated Metrics** | **Value** |
| --- | --- |
| Average windowed ΔG | -31.00 |
| Lowest average windowed ΔG | -66.68 |
| Highest average windowed ΔG | -3.68 |
| Average windowed z-score | -0.43 |
| Lowest average windowed z-score | -3.59 |
| Highest average windowed z-score | 1.65 |
| Average % of windows with a z-score ≤ -1 | 28.99 |
| Lowest % of windows with a z-score ≤ -1 | 0.00 |
| Highest % of windows with a z-score ≤ -1 | 100.00 |
| Average % of windows with a z-score ≤ -2 | 8.60 |
| Lowest % of windows with a z-score ≤ -2 | 0.00 |
| Highest % of windows with a z-score ≤ -2 | 100.00 |
| Average numer of motifs | 3.92 |
| Lowest number of motifs | 0.00 |
| Highest number of motifs | 181.00 |
| Average sequence length | 2194.10 |
| Lowest sequence length | 120.00 |
| Highest sequence length | 104783.00 |
| Average number of windows | 2075.10 |
| Lowest number of windows | 1.00 |
| Highest number of windows | 104664.00 |
| Average per nucleotide 5’UTR ΔG z-score | -0.71 |
| Average per nucleotide CDS ΔG z-score | -0.82 |
| Average per nucleotide 3’UTR ΔG z-score | -0.92 |

**Table S1.** Average, highest, and lowest values for all transcriptome metrics; and the regional average z-scores for all transcriptome metrics
