# Supplementary material for: Expansion of the RNAStructuromeDB to include secondary structural data spanning the human protein-coding transcriptome
Source: Sci Rep. 2022 Aug 25;12:14515. doi: 10.1038/s41598-022-18699-3 (PMC9403969; doi:10.1038/s41598-022-18699-3)
Supplement: Supplementary file 1 — Supplementary Information. [file 41598_2022_18699_MOESM1_ESM.zip › Supplemental/Table S2.docx]

| **Group** | **# of Genes in each Group** | **# of Genes Analyzed** | **Description of group** |
| --- | --- | --- | --- |
| Tissue enriched expression | 10992 | 10383 | Genes that display elevated expression in at least one of the analyzed tissues. |
| Detected in all | 8839 | 8747 | Genes that have detectable levels (nTPM≥1 or transcription frequency≥1) of transcribed mRNA molecules in all tissues. |
| Detected in many | 5956 | 5684 | Genes that have detectable levels (nTPM≥1 or transcription frequency≥1) of transcribed mRNA molecules in at least one third but not all tissues. |
| Detected in some | 3368 | 3124 | Genes that have detectable levels (nTPM≥1 or transcription frequency≥1) of transcribed mRNA molecules in more than one but less than one third of tissues. |
| Detected in single | 1062 | 938 | Genes that have detectable levels (nTPM≥1 or transcription frequency≥1) of transcribed mRNA molecules in a single tissue. |
| Tissue enriched genes | 3107 | 2907 | Genes that display at least four-fold higher mRNA level in a particular tissue compared to any other tissue. |
| Group enriched genes | 1691 | 1596 | Genes that display at least four-fold higher average mRNA level in a group of 2-5 tissues compared to any other tissue. |
| Tissue Enhanced genes | 6194 | 5880 | Genes that display at least four-fold higher mRNA level in a particular tissue compared to the average level in all other tissues. |
| HKGs | 8839 | 8747 | Genes of house-keeping genes that are crucial for the maintenance of basic cellular function. |
| Regulatory TFs | 1490 | 1469 | Genes of transcription factors that are regulatory proteins known to bind to consensus DNA sequences and activate transcription. |

**Table S2**. Expression dataset groups, number of analyzed genes, and a description of the group
