# Supplementary material for: Expansion of the RNAStructuromeDB to include secondary structural data spanning the human protein-coding transcriptome
Source: Sci Rep. 2022 Aug 25;12:14515. doi: 10.1038/s41598-022-18699-3 (PMC9403969; doi:10.1038/s41598-022-18699-3)
Supplement: Supplementary file 1 — Supplementary Information. [file 41598_2022_18699_MOESM1_ESM.zip › Supplemental/Table S3.docx]

| ENST ID | ENST00000306434.8 |
| --- | --- |
| Avg windowed MFE (kcal/mol) | -27.27 |
| Avg windowed z-score | -0.52 |
| # of Windows | 2698 |
| # of ZS windows ≤ -1 | 945 |
| % of ZS windows ≤ -1 | 35.03 |
| # of ZS windows ≤ -2 | 301 |
| % of ZS windows ≤ -2 | 11.16 |
| Sequence Length | 2817 |
| # of Motifs with z-score ≤ -2 | 6 |
| 5'UTR nucleotide positions | 1 - 120 |
| 5'UTR average per nucleotide z-score | -0.40 |
| CDS nucleotide positions | 121 - 1308 |
| CDS average per nucleotide z-score | -0.87 |
| 3'UTR nucleotide positions | 1309 - 2817 |
| 3'UTR average per nucleotide z-score | -1.07 |
| Stem Loop A overall z-score | -2.83 |
| Stem Loop B overall z-score | -3.79 |
| Stem Loop C overall z-score | -4.09 |
| Stem Loop D overall z-score | -3.68 |
| Stem Loop E overall z-score | -2.87 |
| Stem Loop F overall z-score | -2.45 |

**Table S3.** MAT2A ScanFold metrics for the entire transcript, regional analysis, and stem loop structures of the 3’UTR.
