# Supplementary material for: Expansion of the RNAStructuromeDB to include secondary structural data spanning the human protein-coding transcriptome
Source: Sci Rep. 2022 Aug 25;12:14515. doi: 10.1038/s41598-022-18699-3 (PMC9403969; doi:10.1038/s41598-022-18699-3)

MAT2A_M2.stockholm_1

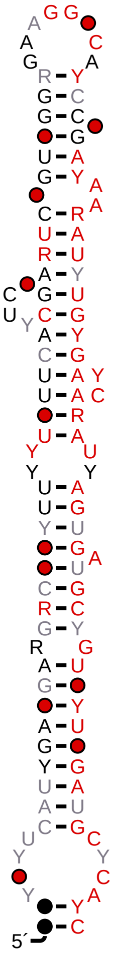

MAT2A_M3.stockholm_1

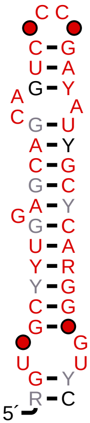

MAT2A_M4.stockholm_1

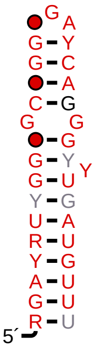

MAT2A_M5.stockholm_1

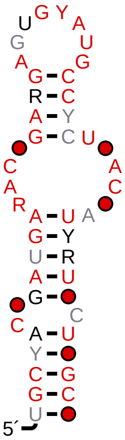

MAT2A_M6.stockholm_1

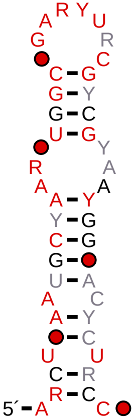

M7.stockholm_1

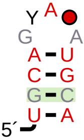

MAT2A_M8.stockholm_1

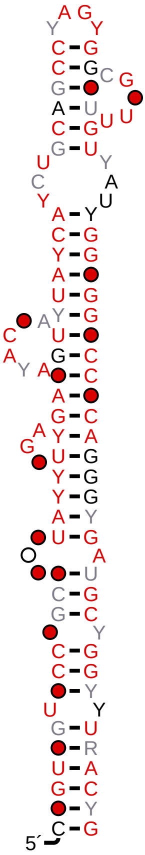

MAT2A_M9.stockholm_1

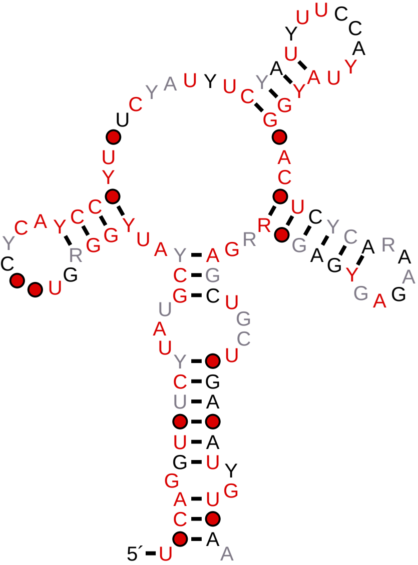

M10.stockholm_1

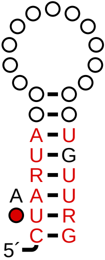

MAT2A_M13.stockholm_1

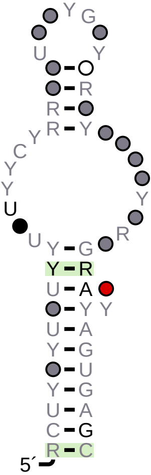

MAT2A_M14.stockholm_1

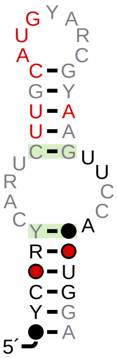

MAT2A_M15.stockholm_1

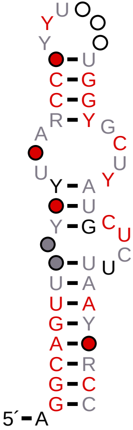

MAT2A_M16.stockholm_1

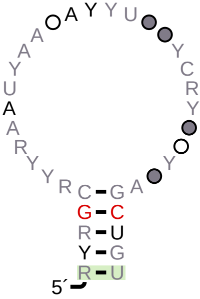

M17.stockholm_1

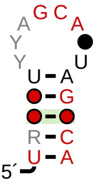

M19.stockholm_1

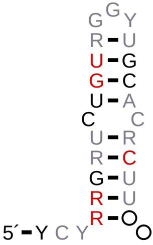

M20.stockholm_1

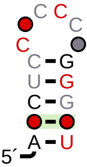

M24.stockholm_1

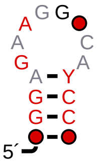

M30.stockholm_1

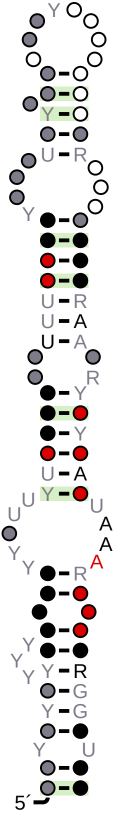

SL_A.stockholm_1

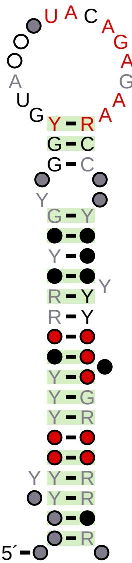

SL_B.stockholm_1

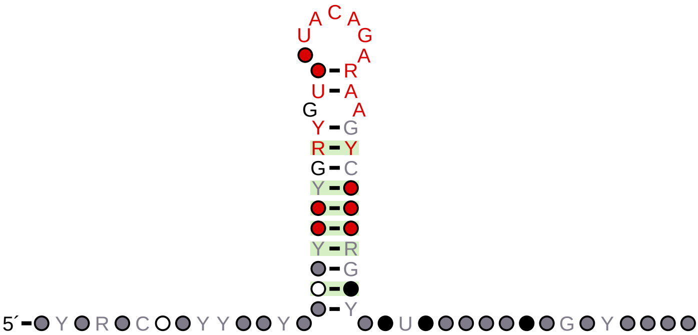

SL_C.stockholm_1

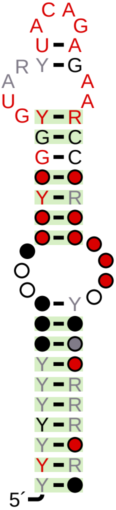

SL_D.stockholm_1

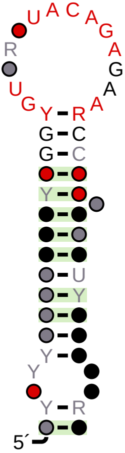

SL_E.stockholm_1

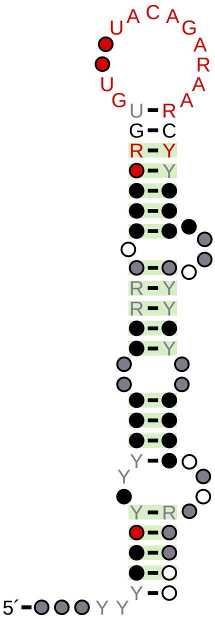

SL_F.stockholm_1

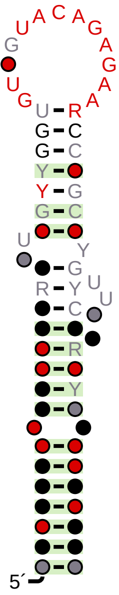

Supplement: Supplementary file 1 — Supplementary Information. [file 41598_2022_18699_MOESM1_ESM.zip › Supplemental/File S6.zip!/File S6/CaCo/MAT2A.MinCov4CaCoFold.pdf]
